# Concurrent Atezolizumab Plus Bevacizumab and High-Dose External Beam Radiotherapy for Highly Advanced Hepatocellular Carcinoma

**DOI:** 10.1093/oncolo/oyae048

**Published:** 2024-03-26

**Authors:** Chung-Wei Su, Wei Teng, Eric Yi-Liang Shen, Bing-Shen Huang, Po-Ting Lin, Ming-Mo Hou, Tsung-Han Wu, Din-Li Tsan, Chia-Hsun Hsieh, Ching-Ting Wang, Pei-Mei Chai, Chun-Yen Lin, Shi-Ming Lin, Chen-Chun Lin

**Affiliations:** Department of Gastroenterology and Hepatology, Chang Gung Memorial Hospital, Linkou Medical Center, Taoyuan, Taiwan; College of Medicine, Chang Gung University, Taoyuan, Taiwan; Department of Gastroenterology and Hepatology, Chang Gung Memorial Hospital, Linkou Medical Center, Taoyuan, Taiwan; College of Medicine, Chang Gung University, Taoyuan, Taiwan; College of Medicine, Chang Gung University, Taoyuan, Taiwan; Department of Radiation Oncology and Proton Therapy Center, Chang Gung Memorial Hospital, Linkou Medical Center, Taoyuan, Taiwan; Clinical Metabolomics Core Lab, Chang Gung Memorial Hospital, Linkou Medical Center, Taoyuan, Taiwan; College of Medicine, Chang Gung University, Taoyuan, Taiwan; Department of Radiation Oncology and Proton Therapy Center, Chang Gung Memorial Hospital, Linkou Medical Center, Taoyuan, Taiwan; Department of Gastroenterology and Hepatology, Chang Gung Memorial Hospital, Linkou Medical Center, Taoyuan, Taiwan; College of Medicine, Chang Gung University, Taoyuan, Taiwan; College of Medicine, Chang Gung University, Taoyuan, Taiwan; Division of Hematology-Oncology, Department of Internal Medicine, Chang Gung Memorial Hospital, Linkou Medical Center, Taoyuan, Taiwan; College of Medicine, Chang Gung University, Taoyuan, Taiwan; Department of General Surgery, Chang Gung Memorial Hospital, Linkou Medical Center, Taoyuan, Taiwan; College of Medicine, Chang Gung University, Taoyuan, Taiwan; Department of Radiation Oncology and Proton Therapy Center, Chang Gung Memorial Hospital, Linkou Medical Center, Taoyuan, Taiwan; College of Medicine, Chang Gung University, Taoyuan, Taiwan; Division of Hematology-Oncology, Department of Internal Medicine, Chang Gung Memorial Hospital, Linkou Medical Center, Taoyuan, Taiwan; Division of Hematology-Oncology, Department of Internal Medicine, New Taipei Municipal Tucheng Hospital (Built and Operated by Chang Gung Memorial Hospital), New Taipei, Taiwan; College of Medicine, Chang Gung University, Taoyuan, Taiwan; Department of Nursing, Chang Gung Memorial Hospital, Linkou Medical Center, Taoyuan, Taiwan; College of Medicine, Chang Gung University, Taoyuan, Taiwan; Department of Nursing, Chang Gung Memorial Hospital, Linkou Medical Center, Taoyuan, Taiwan; Department of Gastroenterology and Hepatology, Chang Gung Memorial Hospital, Linkou Medical Center, Taoyuan, Taiwan; College of Medicine, Chang Gung University, Taoyuan, Taiwan; Department of Gastroenterology and Hepatology, Chang Gung Memorial Hospital, Linkou Medical Center, Taoyuan, Taiwan; College of Medicine, Chang Gung University, Taoyuan, Taiwan; College of Medicine, Chang Gung University, Taoyuan, Taiwan; Department of Gastroenterology and Hepatology, New Taipei Municipal Tucheng Hospital (Built and Operated by Chang Gung Memorial Hospital), New Taipei, Taiwan

**Keywords:** hepatocellular carcinoma, portal vein thrombosis, atezolizumab plus bevacizumab, radiotherapy, toxicity

## Abstract

**Background:**

Atezolizumab plus bevacizumab (atezo-bev) has been recommended for advanced hepatocellular carcinoma (HCC). High-dose external beam radiotherapy (RT) is recognized for its excellent local tumor control. The efficacy and safety of concurrent atezo-bev with RT for highly advanced HCC has been minimally explored.

**Methods:**

In this preliminary retrospective study, we assessed patients with highly advanced HCC, characterized by Vp4 portal vein thrombosis or tumors exceeding 50% of liver volume, who received concurrent atezo-bev and RT (group A). Group A included 13 patients who received proton radiation at a dose of 72.6 GyE in 22 fractions, and one patient who received photon radiation at a dose of 54 Gy in 18 fractions. This group was compared with 34 similar patients treated atezo-bev alone as a control (group B). The primary objectives were to evaluate the objective response rate (ORR), overall survival (OS), and safety.

**Results:**

Baseline characteristics were similar between groups, except for a higher incidence of Vp4 portal vein thrombosis in group A (78.6% vs. 21.4%, *P* = .05). Group A achieved a higher ORR (50.0% vs. 11.8%, *P* < .01) and a longer OS (not reached vs. 5.5 months, *P* = .01) after a median follow-up of 5.2 months. Multivariate analysis indicated that concurrent RT independently favored longer OS (hazard ratio: 0.18; 95% CI, 0.05-0.63, *P* < .01). Group A did not increase any grade adverse events (78.6% vs. 58.8%, *P* = .19) or severe adverse events of grade ≥ 3 (14.3% vs. 14.7%, *P* = .97) compared to group B.

**Conclusions:**

The concurrent high-dose external beam radiotherapy appears to safely enhance the effectiveness of atezolizumab plus bevacizumab for highly advanced patients with HCC. Further studies are warranted to confirm these findings.

## Teaser

This preliminary retrospective study assessed patients with highly advanced hepatocellular carcinoma, exploring the efficacy and safety of concurrent atezolizumab plus bevacizumab with high-dose external beam radiotherapy for these patients.

**Implications for Practice**

Highly advanced hepatocellular carcinoma (HCC), characterized by major portal vein thrombosis or tumors exceeding 50% of liver volume, has an extremely poor outcome. The combination of atezolizumab plus bevacizumab (atezo-bev) is recommended for advanced HCC, and high-dose external beam radiotherapy (RT) can provide excellent local tumor control. In this study, the combination of atezo-bev with concurrent RT was found to be safe. This combination demonstrated a favorable outcome in terms of tumor response, intrahepatic tumor control, preserved liver function, and the potential to prolong overall survival.

## Introduction

Hepatocellular carcinoma (HCC) represents a major global healthcare burden. Predictions indicate that the number of new cases and HCC-related mortality will increase by 55% over the next 20 years.^1^ Atezolizumab plus bevacizumab (atezo-bev) has been recommended as one of the first-line treatments for unresectable HCC.^2^ Highly advanced HCC, particularly cases with total portal vein thrombosis or a significant tumor volume, is considered a critical poor prognostic factor.^3^ The IMbrave 150 trial included patients with highly advanced HCC, and exploratory data revealed that atezo-bev increased the objective response rate (ORR) and reduced the hazard ratio for overall survival (OS) to 0.62 in high-risk advanced patients with Vp4 portal vein thrombosis when compared to sorafenib.^4^ However, despite the ORR increasing to 23%, the OS remained at only 7.6 months.

High-dose external beam radiation therapy (RT) can offer excellent local control for intrahepatic HCC.^5^ Stereotactic body radiation therapy (SBRT) and proton beam radiation (PBT) have demonstrated local tumor control rates up to 90% with minimal damage to the surrounding non-tumor liver tissue.^6^ Clinical guidelines recommended external beam RT for the treatment of multifocal or unresectable HCC as well as those with macrovascular invasion.^7^ A recent phase I study has suggested that combining immunotherapy with RT may yield better outcomes compared to immunotherapy alone.^8^ Preliminary studies have shown a well-tolerated safety profile when combining atezo-bev and RT for HCC treatment.^9,10^ Our previous research indicated that PBT can be safely combined with immune checkpoint inhibitors, achieving an ORR of up to 50% for advanced HCC.^11^ However, to date, no study has specifically focused on the safety and efficacy of high-dose RT combined with atezo-bev for treating the highly advanced HCC with Vp4 portal vein thrombosis or large liver tumors exceeding 50% of the liver. Therefore, this retrospective comparative study aims to investigate the ORR, overall survival, and safety of SBRT or PBT when combined with atezo-bev in comparison to atezo-bev alone for highly advanced HCC patients.

## Materials and Methods

### Patient Recruitment

We conducted a review of 184 consecutive patients with unresectable hepatocellular carcinoma (HCC) who received atezo-bev treatment between September 2020 and December 2022 at both a tertiary medical center and a secondary referral hospital in Taiwan. This study specifically focused on 14 patients with advanced HCC who presented with complication of Vp4 portal vein thrombosis or tumor occupancy more than 50% of liver volume and received concurrent atezo-bev and high-dose RT (group A). Additionally, we included another 34 similar patients with advanced HCC with Vp4 portal vein thrombosis or tumor occupancy more than 50% of liver volume who received atezo-bev alone as a control group (group B). The diagnosis of HCC was established based on either pathology findings or imaging criteria defined by the American Association for the Study of Liver Disease.^12^ All the 48 included patients had either major portal vein thrombosis or a high tumor burden more than 50% of the liver volume. Our study excluded patients who fell under the criteria: Child-Pugh class B or C, Eastern Cooperative Oncology Group (ECOG) performance status more than 2, recent severe systemic infection, uncontrol gastrointestinal bleeding, without evaluable baseline imaging, or other malignancies. The objectives of the study were ORR, OS, and safety.

Multidisciplinary meetings, involving hepatologist, surgeon, medical oncologist, radiational oncologist, radiologist, and pathologist, were conducted to discuss the treatment details prior to shared decision making between patients and clinical doctors. The study received approval from the institutional review board of Chang Gung Medical Foundation (IRB No. 202300597B0), and written informed consent was waived due to the retrospective design.

### Treatment and Follow-Up Protocol

All patients received intravenous atezolizumab 1200 mg and bevacizumab 5-15 mg/kg every 3 weeks until one of the following criteria was met: disease progression, development of adverse reactions that led to intolerance, or a patient’s decision to discontinue treatment. Patients in group A patients underwent concurrent high-dose RT, which commenced within 2 weeks of the initial administration of atezo-bev. For high-dose RT treatment planning, 13 patients received proton therapy and one used SBRT treatment. Each patient underwent a contrast-enhanced 4-dimensional computed tomography (CT) simulation with abdominal compression. Magnetic resonance simulation was performed for patients who were planning to receive proton therapy or SBRT therapy. The internal target volume (ITV) was expanded according to the gross target volume on each respiratory phase of 4-dimensioinal CT. The planning target volume (PTV) was calculated by expanding the ITV by 5-7 mm in each direction. Image-guided RT using daily cone beam computed tomography (CBCT) was recommended. It was also suggested that patients fast for 4 hours prior to CT simulations and each treatment, if tolerable. The RT approach in this study was specifically targeted toward portal vein thrombosis and aimed to cover the major target tumors as extensively as possible. The coverage for PTV was primarily constrained by the proximity to hollow organs and the liver’s tolerance. When the PTV was close to the gastrointestinal tract, adherence to GI constraints was prioritized. Furthermore, the coverage of the PTV was constrained by considerations of liver tolerance, aiming to preserve maximal normal liver tissue. Despite these limitations, we ensure that the minimum prescribed dose to the PTV is maintained at no less than 60% of the prescribed dose. The irradiation scope in the RT planning was adjusted during concurrent therapy based on repeat MRI scans, especially if there was any evidence of target tumor displacement. Adaptive planning was essential in response to anatomical changes during treatment, which could lead to suboptimal target coverage or unnecessary irradiation of organs at risk. The decision to modify radiation fields for adaptive plans relied on multiple factors, such as patient positioning consistency, diaphragmatic movement, and anatomical changes seen in CBCT, CT, and MRI simulations. MRI simulations, while carried out in the same positioning, were performed using an independent machine. The frequency of these revisions was primarily guided by physician preference and clinical necessity. Nonetheless, CBCT was undertaken regularly, and repeat CT simulations and adaptive planning were conducted at least once during radiotherapy.

The prescribed dose of proton therapy was 66 Gy equivalent (GyE) in 10 fractions for patients whose ITV was located more than 2 cm away from the hilar region or the gastrointestinal tract, including the esophagus, stomach, and small and large intestines. Patients with ITV located <2 cm from the hilar region or gastrointestinal tract received a prescription of 72.6 GyE in 22 fractions. The relative biological effectiveness value for proton therapy was set at 1.1. For SBRT therapy, the goal was to achieve a biologically effective dose (BED_10_) higher than 55 Gy was recommended. The most common prescribed dose was 45-60 Gy in 15-20 fractions, depending on the volume of normal liver exposed to radiation. The radiation dose constraints for organs at risk were consistent for all patients receiving radiation therapy (Supplementary Table S1).

Before treatment and at intervals of 2-4 weeks following treatment, we measured serum markers including liver and renal function, as well as serum alpha-fetoprotein levels every 3-4 weeks. Additionally, we conducted image evaluations using dynamic CT or magnetic resonance imaging (MRI) every 2-3 months from the initiation of treatment. Radiological responses were classified into complete response (CR), partial response (PR), stable disease (SD), or progressive disease (PD) according to Modified Response Evaluation Criteria in Solid Tumors (mRECIST). All treatment-related adverse events (TRAEs) were documented following the Common Terminology Criteria for Adverse Events v5.0 (NCI CTCAE; version 5.0). Radiation-induced liver disease (RILD) was defined as follows: classic RILD, characterized by anicteric hepatomegaly and ascites with alkaline phosphatase levels exceeding twice the upper limit of normal; non-classic RILD, defined as bilirubin levels exceeding 3 times the upper limit of normal or aspartate aminotransferase (AST) or alanine aminotransferase (ALT) levels exceeding 5 times the upper limit of normal.^13^

### Statistical Analysis and Definitions

Descriptive data with and without normal distribution are presented as mean ± standard deviation and median (interquartile range, IQR), respectively. Differences between groups for variables with normal distribution were assessed using an independent Student’s *t* test, whereas the Mann-Whitney U test was applied for variables without normal distribution. The differences in categorical variables between the 2 groups were compared using either the Chi-square test or Fisher’s exact test.

Progression-free survival (PFS) was defined as the time from the date of the first atezo-bev treatment to radiological PD or death, while OS was calculated from the first atezo-bev treatment to the date of death. Time to progression (TTP) referred to the period of the initial atezo-bev treatment to radiology progression. The time to progression to ALBI grade 3 was calculated from the initial atezo-bev treatment until the occurrence of ALBI grade 3 or until censoring at the 6-month mark. For patients who were alive without radiological PD, we censored at the date of the last contact or data cutoff date, December 31, 2022. PFS, OS, TTP were estimated using Kaplan-Meier method and subgroup comparisons were made using the log-rank test. Logistic regression and cox-regression models were performed to determine predictive factors of responders, PFS and OS. A 2-tailed *P*-value of <.05 was defined as statistically significant. We conducted all statistical analyses using the following software programs: SAS version 9.4, SPSS software (release Version 22.0, IBM Corp., Armonk, NY, USA), and STATA (STATA version 14.0; StataCorp, College Station, TX).

## Results

### Baseline Characteristics and Treatment Dose

The baseline characteristics of both groups are presented in Table 1. There were no significant differences between the 2 groups in baseline variables including age, gender, etiology, ECOG PS, ALBI grade, BCLC stage, and extrahepatic spreading. However, group A had a higher proportion of VP4 portal vein invasion compared to group B (78.6% vs. 21.4%, *P* = .0462). Notably, the follow-up periods and the duration of atezo-bev treatment were significantly longer in group A than in group B (median follow-up periods: 7.3 months vs. 4.4 months, *P* = .0412; median treatment duration: 4.5 months vs. 3.0 months, *P* = .0107). In group A, the radiation dose was 72.6 GyE in 22 fractions for 13 proton radiation patients and 54 Gy in 18 fractions for the photon radiation patient.

**Table 1.** Baseline characteristics of the patients with highly advanced HCC received atezo-bev treatment.

Variables	Group A, *N* = 14 (atezo-bev with RT)	Group B, *N* = 34 (atezo-bev alone)	*P*-value
Age	62.0 ± 14.4	61.1 ± 11.5	.8653
≥60	9 (64.3)	20 (58.8)	.7250
Male gender	11 (78.6)	24 (70.6)	.5716
Etiology			
Virus	13 (92.9)	28 (82.4)	.3486
Non-virus	1 (7.1)	6 (15.6)	
ECOG			
0	8 (57.1)	21 (61.8)	.7660
1 or 2	6 (42.9)	13 (38.2)	
Modified ALBI grade^†^			
I or IIa	8 (61.5)	20 (60.6)	.9535
IIb	5 (38.5)	13 (39.4)	
BCLC stage			
B	3 (21.4)	7 (20.6)	.9480
C	11 (78.6)	27 (79.4)	
Maximum tumor size (cm)	10.6 (IQR: 9.0-12.0)	11.3 (IQR: 8.6-14.0)	.5176
≥10 cm	9 (64.3)	22 (64.7)	.9779
Beyond up-to-11 criteria	11 (78.6)	28 (82.4)	.7603
Portal vein tumor thrombosis			
VP stage 0-3	3 (21.4)	21 (78.6)	.0462
VP stage 4	11 (78.6)	13 (21.4)	
Occupied > 50% liver volume	10 (71.4)	28 (82.4)	.3969
Extra-hepatic metastasis	4 (28.6)	12 (35.3)	.6534
AFP (ng/mL)			
>400	8 (57.1)	18 (52.9)	.7906
Prior locoregional therapy^†^^,^^‡^	3 (21.4)	16 (47.1)	.0989
Systemic treatment			
First line	12 (85.7)	22 (64.7)	.1455
≥2nd line	2 (14.3)	12 (35.3)	

^†^ALBI missing for one patient in atezo-bev alone and one patient in atezo-bev with RT.

^‡^One patient in group B had history of proton beam therapy as prior HCC treatment.

Abbreviations: HCC, hepatocellular carcinoma; atezo-bev, atezolizumab plus bevacizumab; RT, external beam radiation therapy; ECOG, Eastern Cooperative Oncology Group; ALBI, albumin-bilirubin index; BCLC, Barcelona Clinic Liver Cancer; AFP, α-fetoprotein.

### Treatment Response

Radiological responses were summarized in Table 2. In group A (*n* = 14), 7 patients achieved an objective response, with one achieving CR. In group B (*n* = 34), only 4 patients achieved PR without any patients achieving CR. Group A exhibited significantly higher ORR and DCR compared to group B (ORR: 50.0% vs. 11.8%, *P* = .0042; DCR: 78.6% vs. 47.1%, *P* = .0455). The ORRs of group A were significantly better than those of group B in treatment-naive patients (first-line: 58.3% vs 11.8%, *P* = .0140) and those of the patients with large tumor volume occupied more than 50% of the liver (40.0% vs 8.7%, *P* = .0321). However, there was no significant difference between the 2 groups in terms of VP4 patients (36.4% vs. 30.0%, *P* = .7574). In logistic regression model analysis, 2 factors were identified as independent factors associated with tumor response (Supplementary Table S2). A huge tumor exceeding 50% of the liver was a negative factor for tumor response (adjusted odds ratio = 0.173, 95% CI: 0.030-0.994; *P* = .0492), and receiving concurrent RT was a positive factor for tumor response (adjusted odds ratio = 6.138 95% CI: 1.236-30.494; *P* = .0265).

**Table 2.** Treatment outcome for patients with highly advanced HCC received atezo-bev treatment.

	Group A, *N* = 14 atezo-bev with RT	Group B, *N* = 34 atezo-bev alone	*P*-value
Best response by mRECIST			
Complete response	1 (7.1)	0 (0.0)	
Partial response	6 (42.9)	4 (11.8)	
Stable disease	4 (28.6)	12 (35.3)	
Progressive disease	3 (21.4)	12 (35.3)	
No image evaluation	1 (7.1)	5 (14.7)	
Objective response rate	7 (50.0)	4 (11.8)	.0042
Disease control rate	11 (78.6)	16 (47.1)	.0455
Observation period (months)	7.3 (IQR: 4.2-12.0)	4.4 (IQR: 2.7-7.8)	.0412
Treatment duration (months)	4.5 (IQR: 4.2-7.6)	3.0 (IQR: 2.1-4.8)	.0107
Atezo-bev treatment course	6 (IQR: 4-7)	3 (IQR: 2-6)	.0039
Mortality	3 (21.4)	20 (58.8)	.0184
All grade AEs	11 (78.6)	20 (58.8)	.1935
Severe AE (≥grade 3)	2 (14.3)	5 (14.7)	.9701

Abbreviations: HCC, hepatocellular carcinoma; atezo-bev, atezolizumab plus bevacizumab; RT, external beam radiation therapy; mRECIST, modified Response Evaluation Criteria in Solid Tumors; AE, adverse event.

**Figure 1.**
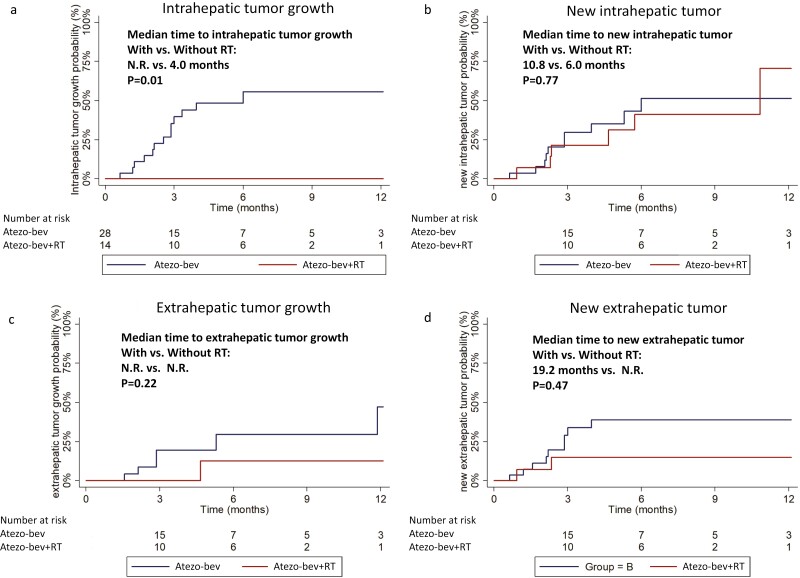
Kaplan-Meier failure function of time to progression for highly advanced patients received atezo-bev. (**a**) Intrahepatic tumor growth, (**b**) new intrahepatic tumor, (**c**) extrahepatic tumor growth, and (**d**) new extrahepatic tumor growth. atezo-bev, atezolizumab plus bevacizumab; RT, external beam radiation therapy.

Group A had a significantly longer median time to intrahepatic tumor growth (IHG) compared with group B (not reached vs 4.0 months; *P* = .0057) (Fig. 1a). However, there was no significant differences between 2 groups in the median time to new intrahepatic tumor (NIH: 10.8 months vs 6.0 months; *P* = .7728; Fig. 1b), extrahepatic tumor growth (EHG: not reached vs not reached, *P* = .2197; Fig. 1c), or new extrahepatic tumor (NEH: 19.2 months vs not reached, *P* = .4737; Fig. 1d). At the data cutoff date, 4 (28.6%) patients in group A and 3 (8.8%) patients in group B were still receiving atezo-bev treatment.

### Overall Survival

**Figure 2.**
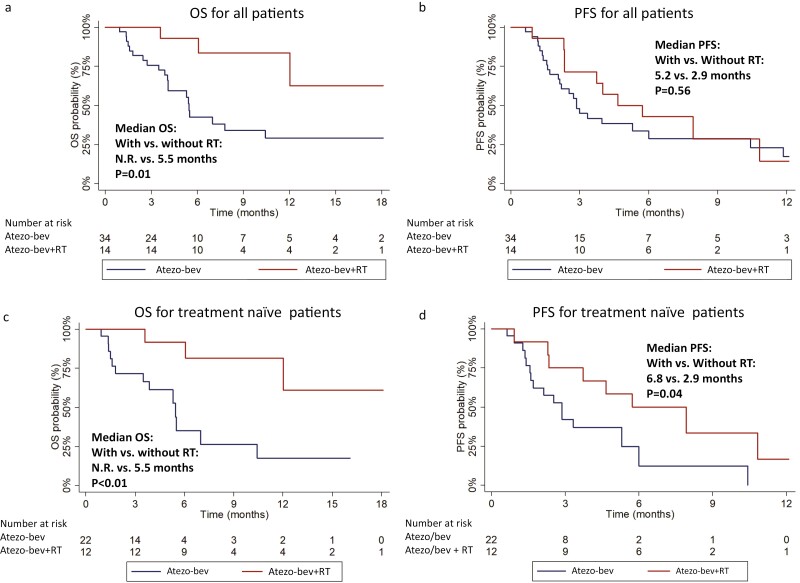
Kaplan-Meier survival function for highly advanced patients received atezo-bev. (**a**) OS for overall patients, (**b**) PFS for overall patients, (**c**) OS for atezo-bev as first-line therapy patients, and (**d**) PFS for atezo-bev as first-line therapy patients. atezo-bev, atezolizumab plus bevacizumab; RT, external beam radiation therapy; OS, overall survival; PFS, progression-free survival.

At the data cutoff date, 3 patients (21.4%) in group A died, and 10 patients (71.4%) had overall disease progression, while in group B, 20 patients (58.8%) died, and 24 patients (70.6%) experienced the overall disease progression. Regarding OS, the cumulative survival rates at 6 months, 12 months, 18 months were 93.9%, 62.7%, and 62.7% in group A, and 42.5%, 34.0%, and 29.1% in group B, respectively. Group A had a significantly longer OS than group B (median OS: not reached vs. 5.5 months, *P* = .0131; Fig. 2a). However, no significant difference was observed between 2 groups in terms of PFS (median PFS: 5.2 months vs 2.9 months, *P* = .5625; Fig. 2b). There was no significant difference in the proportions of sequential therapies after atezo-bev between the 2 groups (group A vs group B, 35.7% vs. 47.0%, *P* = .4604; Supplementary Table S3). Among the 34 patients received atezo-bev as first-line systemic treatment, patients in group A had significant longer OS and PFS than those in group B (median OS: not reached vs 5.5 months, *P* = .0081; median PFS: 6.8 months vs 2.9 months, *P* = .0425) (Fig. 2c and 2d).

The factors predicting PFS and OS were analyzed using a Cox-regression model. For PFS, univariate analysis suggested associations with BCLC stage, VP4 complicated with tumor exceeding 50% of liver, extrahepatic metastasis, AFP ≥ 400 ng/mL, and prior locoregional therapy in these patients with highly advanced HCC. However, multivariate analysis did not identify any independent predictor (Supplementary Table S4). For OS, univariate analysis suggested that the etiology of underlying liver disease, BCLC stage, prior locoregional therapy, and concurrent RT might be predictors. In the multivariate analysis, concurrent RT emerged as the only independent factor (adjusted HR = 0.180, 95% CI: 0.051-0.632; *P* = .0075), while prior locoregional therapy showed a trend toward longer OS (adjusted HR = 0.351 95% CI: 0.121-1.021; *P* = .0546; Table 3).

**Table 3.** Predictors for overall survival in the patients with highly advanced HCC received atezo-bev treatment.

Variables	All (*N* = 48)	Median OS (95% CI)	Crude HR (95% CI)	*P-*value	Adjust HR (95% CI)	*P*-value
Age (years)						
<60	19 (39.6)	7.8000	Referent			
≥60	29 (60.4)	10.4333	0.930 (0.407-2.125)	0.8630		
Gender						
Female	13 (27.1)	5.5000	Referent			
Male	35 (72.9)	7.8000	1.034 (0.406-2.632)	0.9435		
Etiology						
Virus	41 (85.4)	10.4000	Referent		Referent	
Non-virus	7 (14.6)	1.8000	2.379 (0.879-6.439)	0.0881	2.252 (0.801-6.329)	.1236
ECOG						
0	29 (60.4)	12.0333	Referent			
1 or 2	19 (39.6)	7.0000	1.626 (0.668-3.961)	0.2844		
Modified ALBI grade^†^						
I or IIa	28 (60.9)	10.4333	Referent			
IIb or III	18 (39.1)	5.5000	1.269 (0.533-3.017)	0.5904		
BCLC stage						
B	10 (20.8)	NR	Referent		Referent	
C	38 (79.2)	6.0667	3.539 (0.828-15.129)	0.0882	2.488 (0.515-12.018)	.2566
Beyond up-to-11 criteria						
No	9 (18.8)	12.0333	Referent			
Yes	39 (81.3)	7.8000	1.098 (0.404-2.987)	0.8544		
High-risk factors						
No	34 (70.8)	10.4333	Referent			
Yes	14 (29.2)	5.4667	1.614 (0.680-3.831)	0.2774		
Extrahepatic metastasis						
No	32 (66.7)	10.4333	Referent			
Yes	16 (33.3)	5.4667	1.242 (0.525-2.940)	0.6219		
AFP (ng/mL)						
<400	22 (45.8)	10.4333	Referent			
≥400	26 (54.2)	5.4667	1.779 (0.766-4.129)	0.1802		
Prior locoregional therapy						
No	29 (60.4)	5.5000	Referent		Referent	
Yes	19 (39.6)	NR	0.431 (0.169-1.098)	0.0778	0.351 (0.121-1.021)	.0546
First-line systemic treatment						
No	14 (29.2)	7.8000	Referent			
Yes	34 (70.8)	7.0000	1.164 (0.478-2.834)	0.7373		
With radiotherapy						
No	34 (70.8)	5.4667	Referent		Referent	
Yes	14 (29.2)	NR	0.242 (0.071-0.816)	0.0222	0.180 (0.051-0.632)	.0075

^†^ALBI missing for one patient in atezo-bev alone and one patient in atezo-bev with RT.

Abbreviations: HCC, hepatocellular carcinoma; atezo-bev, atezolizumab plus bevacizumab; RT, external beam radiation therapy; ECOG, Eastern Cooperative Oncology Group; ALBI, albumin-bilirubin index; BCLC, Barcelona Clinic Liver Cancer; AFP, alpha-fetoprotein.

### Safety

**Figure 3.**
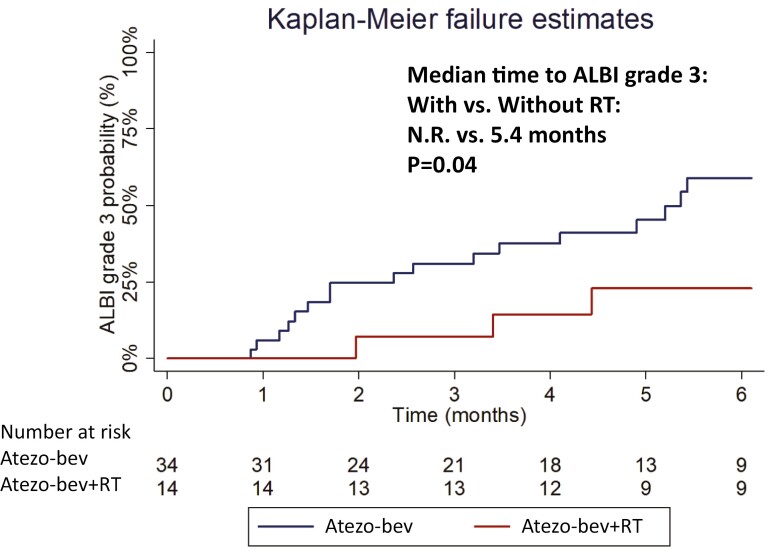
Kaplan-Meier failure function of time to ALBI grade 3 for highly advanced patients received atezo-bev. ALBI, albumin-bilirubin index; atezo-bev, atezolizumab plus bevacizumab; RT, external beam radiation therapy.

All patients in group A successfully completed the RT protocol without discontinuing atezo-bev treatment during concurrent therapy. Among the 14 patients in group A, the frequent TRAEs during or after concurrent RT with atezo-bev were increased AST (35.7%), anorexia or nausea (28.6%), dermatitis (28.6%), fatigue (21.4%), increased ALT (21.4%), hypertension (21.4%), pyrexia (14.3%), proteinuria (14.3%), and radiation-related gastrointestinal bleeding (14.3%). The overall rate of any-grade TRAE in group A patients was 78.6%, which was not significantly higher than 58.8% in group B patients (*P* = .1935). The median time to ALBI grade 3 in group A was significantly longer than in group B (not reached vs 5.4 months, *P* = .0425; Fig. 3). Two patients in the group A experienced grade 3 TRAE including a radiation-related gastrointestinal bleeding and a radiation-related dermatitis. Notably, no patient experienced classic RILD or non-classic RILD. Overall, there was no significant difference rates in severe grade TRAE between the 2 groups (14.7% vs 14.3%, *P* = .9701;Table 4).

**Table 4.** Treatment-related adverse events.

	Group A, *N* = 14 atezo-bev with RT		Group B, *N* = 34 atezo-bev alone	
	Any grade	≥Grade 3	Any grade	≥Grade 3
Any adverse events	11 (78.6)	2 (14.3)	20 (58.8)	5 (14.7)
Aspartate aminotransferase increased	5 (35.7)	0 (0.0)	4 (11.8)	0 (0.0)
Anorexia or nausea	4 (28.6)	0 (0.0)	7 (20.6)	0 (0.0)
Dermatitis	4 (28.6)	1 (7.1)	3 (8.8)	1 (2.9)
Fatigue	3 (21.4)	0 (0.0)	9 (26.5)	0 (0.0)
Alanine aminotransferase increased	3 (21.4)	0 (0.0)	4 (11.8)	0 (0.0)
Hypertension	3 (21.4)	0 (0.0)	2 (5.9)	0 (0.0)
Radiation-related gastrointestinal bleeding	2 (14.3)	1 (7.1)	0 (0.0)	0 (0.0)
Pyrexia	2 (14.3)	0 (0.0)	3 (8.8)	0 (0.0)
Proteinuria	2 (14.3)	0 (0.0)	3 (8.8)	0 (0.0)
Colitis	1 (7.1)	0 (0.0)	3 (8.8)	0 (0.0)
Musculoskeletal pain	1 (7.1)	0 (0.0)	2 (5.9)	0 (0.0)
Headache	1 (7.1)	0 (0.0)	0 (0.0)	0 (0.0)
Infusion reaction	1 (7.1)	0 (0.0)	0 (0.0)	0 (0.0)
Thrombocytopenia	1 (7.1)	0 (0.0)	0 (0.0)	0 (0.0)
Oral ulcer	1 (7.1)	0 (0.0)	0 (0.0)	0 (0.0)
Variceal bleeding	0 (0.0)	0 (0.0)	2 (5.9)	2 (5.9)
Bowel perforation	0 (0.0)	0 (0.0)	1 (2.9)	1 (2.9)
Peptic ulcer bleeding	0 (0.0)	0 (0.0)	1 (2.9)	1 (2.9)
Blood bilirubin increased	0 (0.0)	0 (0.0)	1 (2.9)	0 (0.0)
Alkaline phosphatase increase	0 (0.0)	0 (0.0)	1 (2.9)	0 (0.0)
Hyperthyroidism	0 (0.0)	0 (0.0)	1 (2.9)	0 (0.0)
Anal ulcer bleeding	0 (0.0)	0 (0.0)	1 (2.9)	0 (0.0)
Insomnia	0 (0.0)	0 (0.0)	1 (2.9)	0 (0.0)

Abbreviations: atezo-bev, atezolizumab plus bevacizumab; RT, external beam radiation therapy.

## Discussion

This preliminary study demonstrated the safety and effectiveness of high-dose RT using PBT or SBRT in combination with atezo-bev, as compared to atezo-bev alone, for the treatment of patients with highly advanced HCC. High-dose RT has the potential to enhance the treatment response by up to 50% and possibly extend the survival of patients with highly advanced HCC without increasing the risk of severe complications.

Highly advanced HCC, characterized by portal vein total occlusion (Vp4) or a massive liver tumor mass exceeding 50% of the liver volume, has an extremely poor prognosis.^14,15^ Current guidelines recommend atezo-bev, durvalumab-tremelimumab as first-line systemic therapy, and if these are not feasible, sorafenib, lenvatinib, or durvalumab. However, lenvatinib and durvalumab-tremelimumab may lack sufficient evidence to benefit patients with highly advanced HCC, primarily because the HIMALAYA and REFLECT trials excluded patients with severe Vp4 portal vein thrombosis.^16,17^ The exploratory IMbrave 150 report suggested that atezo-bev may provide a survival benefit for high-risk patients.^18^ However, even with an ORR maintained at 23%, the survival for patients with highly advanced HCC remained at approximately 7.5 months. There is still having an unmet need to enhance the response rate and survival for these patients with high-risk HCC.

High-dose RT using SBRT or PBT has proven to be highly effective in providing excellent local tumor control of intrahepatic tumor.^19,20^ The Asia-Pacific primary liver cancer expert consensus also suggests considering high-dose RT for patients with unresectable HCC.^21^ Our prior report demonstrated that the combination of PBT with anti-PD1/PD-L1 achieved an excellent 1-year infield intrahepatic tumor control rate of 70%-90% and effectively obtained an ORR of 43.8%-61.5%. Moreover, it led to a median OS ranging from 20.1 months to not yet reached for patients of unresectable HCC, under palliative or curative treatment approach.^11^ In a recent preliminary single-arm prospective study, the use of intensity-modulated radiotherapy with doses up to 52-56 Gy combined with atezo-bev achieved an ORR up to 76%.^10^ In our current study, 78.6% of patients had Vp4 portal vein thrombosis, and 71.4% patients had intrahepatic tumor burden exceeding 50% of liver volume in group A. SBRT/PBT was used for palliative purpose with the doses as high as 55-72.9 Gy, covering nearly all intrahepatic tumors, except for small tumors in the contralateral lobe of the liver or those located in extremely high-risk locations, such as the distal portal trunk or superior mesentery vein. Even in these cases, we demonstrated that SBRT or PBT combined with atezo-bev significantly increased the ORR to 50% for highly advanced HCC. Furthermore, we showed that SBRT/PBT combined with atezo-bev effectively delayed the progression of intrahepatic tumors. Due to the higher ORR and effective local tumor control of the major intrahepatic tumor, patients may benefit from atezo-bev plus RT treatment in terms of their survival, especially those with bulky tumors or extensive macrovascular invasion.

On the other hands, we did not observe any clear obvious abscopal effect in the combination of SBRT/PBT and atezo-bev for highly advanced HCC. Our findings indicated that this combination did not effectively reduce the incidence of new tumor recurrence or extrahepatic tumor growth when compared to patients receiving atezo-bev alone. Possible reasons for this include a limited number of cases to demonstrate subtle effects. The abscopal effect remains a topic to debate in immunotherapy combined with RT for HCC therapies because certain mechanisms, such as ATR-mediated CD47 and PD-L1 upregulation, have been proposed as potential constraints on radiation-induced immune activation.^22^ The absence of a clear abscopal effect resulted in not significantly improved tumor control in the outfield of SBRT/PBT. This may primarily explain why the combination of SBRT/PBT with atezo-bev only achieved a non-significantly better PFS when compared to patients receiving atezo-bev alone for patients with highly advanced HCC.

The safety profile of the combination of SBRT/PBT with atezo-bev did not increase the risks of any-grade or grade 3 or higher TRAE when compared to atezo-bev alone for highly advanced HCC. In the SBRT/PBT combination with atezo-bev group, TRAE with grade 1/2 elevation of serum AST or ALT levels were slightly higher than the atezo-bev group, but no severe hepatitis events or RILD occurred after starting therapy. In previous studies, the authors reported that SBRT did not induce a higher incidence of liver injury.^23^ Several authors suggested that PBT can minimize the damage to the surrounding liver tissue.^24-26^ Therefore, the precise irradiation of liver tumors and with decreased peripheral liver parenchyma damage contributes significantly to delaying liver function reserve downhill to ALBI grade 3. The better preservation of the liver from the SBRT/PBT combination with atezo-bev is considered to support the survival benefits.

Among the patients receiving SBRT/PBT, 3 (21.4%) experienced radiation-related gastrointestinal bleeding, with 2 patients at grade 2 and one patient at grade 3. To minimized the bleeding risk, we have a policy of screening high-risk patients with upper gastrointestinal bleeding through a pre-treatment esophagogastroduodenoscopy. This helps us avoid atezo-bev therapy in patients with recent major bleeding. The radiation dose constraints for all patients receiving radiation therapy were followed according to the protocol to limit the risk of bowel injury. Notably, these radiation-related bleeding TRAE were observed only in the initial cases. The occurring of bleeding TRAE was minimized when PBT planning was revised to frequently adjust the radiation field during therapy, guided by repeated MRI scans to account for target tumor displacement due to the infield response within the tumor. Consequently, the frequent adjustment of the PBT irradiation field also reduces the risk of perforation in adjacent hallow organs. However, a prior study demonstrated a high risk of bowel injury associated with incorporating SBRT in vascular endothelial growth factor inhibitor therapy.^27^ Therefore, the combination of anti-angiogenic therapy with SBRT/PBT still raises substantial concerns regarding the serious complications.

This study has several limitations. Firstly, there might be potential selection bias between 2 groups in the retrospective study. Photon therapy is comprehensively covered by our national insurance, making it accessible to all patients. In contrast, proton therapy, not being covered, required self-funding. This financial aspect might have influenced patient selection for the different types of RT. Due to the small number of patients and the lack of matching of baseline characteristics, including the different proportions of VP4 and prior locoregional therapy, the effect of confounders cannot be adequately reduced. Secondary, the median observation periods were only 7.3 months for study group and 4.4 months for control group. This short observation period is likely due to the extremely poor prognostic features of the enrolled patients, which may lead to an incomplete understanding and underestimation of late toxicity. The majority of patients received PBT in this study, so caution should be exercised when interpreting the safety profile of SBRT. Another limitation is the absence of comparison of comparisons of RT doses and techniques due to the study’s small-scale. Furthermore, optimal timing for the combination with atezo-bev and RT remains unresolved in this study. Thus, there is an urgent need for randomized trials to overcome these limitations.

## Conclusion

Results of this preliminary study suggest that high-dose RT using SBRT or PBT in combination with atezo-bev may hold potential benefits for the treatment of highly advanced HCC. This combination offers a safe approach, with the addition of SBRT or PBT to atezo-bev, that can delay intrahepatic tumor progression and preserve liver function reserve, resulting in an increased objective tumor response and potentially improve overall survival. Further studies are warranted to confirm our findings.

## Supplementary Material

oyae048_suppl_Supplementary_Tables_1-4

## Data Availability

The data that support the findings of this study are available on request from the corresponding author. The data are not publicly available due to privacy or ethical restrictions.
