# Supplementary material for: Concurrent Atezolizumab Plus Bevacizumab and High-Dose External Beam Radiotherapy for Highly Advanced Hepatocellular Carcinoma
Source: Oncologist. 2024 Mar 26;29(7):e922–31. doi: 10.1093/oncolo/oyae048 (PMC11224977; doi:10.1093/oncolo/oyae048)
Supplement: oyae048_suppl_Supplementary_Tables_1-4 [file oyae048_suppl_supplementary_tables_1-4.docx]

**Supplementary table 1. The specific constraints for organs at risk under each protocol**

| **66GyE/10 fractions protocol** | | **72.6GyE/22 fractions protocol** | |
| --- | --- | --- | --- |
| **Location** | **Radiation dose** | **Location** | **Radiation dose** |
| **Cord** | **Dmax < 3300 cGyE** | **Cord** | **Dmax < 3900 cGyE** |
| **Normal Liver** | **Volume**  **Vol. to spare(1 GyE) > 400 cc**  **D400cc < 100 cGyE** | **Normal Liver** | **Volume**  **Vol. to spare(1 GyE) > 400 cc**  **D400cc < 100 cGyE** |
| **Esophagus** | **V38GyE < 0.5cc**  **V34GyE < 5 cc**  **D0.03cc < 50% PD.** | **Esophagus** | **V44GyE < 0.5 cc**  **V39GyE < 5 cc**  **D0.03cc < 65% PD.** |
| **Stomach** | **V38GyE < 0.5cc**  **V34GyE < 5 cc**  **D0.03cc < 50% PD.** | **Stomach** | **V44GyE < 0.5 cc**  **V39GyE < 5 cc**  **D0.03cc < 65% PD.** |
| **Duodenum** | **V38GyE < 0.5cc**  **V34GyE < 5 cc**  **D0.03cc < 50% P.D.** | **Duodenum** | **V44GyE < 0.5 cc**  **V39GyE < 5 cc**  **D0.03cc < 65% P.D.** |
| **Bowel** | **V38GyE < 0.5cc**  **V34GyE < 5 cc**  **D0.03cc < 50% PD.** | **Bowel** | **V44GyE < 0.5 cc**  **V39GyE < 5 cc**  **D0.03cc < 65% PD.** |
| **Rt Kidney** | **V18GyE < 33%** | **Rt Kidney** | **V18GyE < 33%** |
| **Lt Kidney** | **V18GyE < 33%** | **Lt Kidney** | **V18GyE < 33%** |
| **Heart** | **V38GyE < 30%** | **Heart** | **V44GyE < 30%** |
| **Chest Wall** | **V60GyE < 2 cc**  **V36GyE < 20 cc** | **Chest Wall** | **V72GyE < 2cc**  **V46GyE < 20cc** |

**Supplementary table 2. Predictors for objective response in image evaluable patients**

| **Variables** | **Tumor response (N=11)** | **Crude HR (95% CI)** | **p value** | **Adjust HR (95% CI)** | **p value** |
| --- | --- | --- | --- | --- | --- |
| **Age (years)** |  |  |  |  |  |
| **<60** | **4 (22.2)** | **Referent** |  |  |  |
| **≥60** | **7 (29.2)** | **1.441 (0.349-5.948)** | **0.6134** |  |  |
| **Gender** |  |  |  |  |  |
| **Female** | **2 (18.2)** | **Referent** |  |  |  |
| **Male** | **9 (29.0)** | **1.841 (0.331-10.253)** | **0.4861** |  |  |
| **Etiology** |  |  |  |  |  |
| **Virus** | **10 (26.3)** | **Referent** |  |  |  |
| **Non-Virus** | **1 (25.0)** | **0.933 (0.087-10.040)** | **0.9546** |  |  |
| **ECOG** |  |  |  |  |  |
| **0** | **5 (27.8)** | **Referent** |  |  |  |
| **1 or 2** | **6 (25.0)** | **0.867 (0.217-3.461)** | **0.8395** |  |  |
| **Modified ALBI grade** |  |  |  |  |  |
| **I or IIa** | **8 (33.3)** | **Referent** |  |  |  |
| **IIb or III** | **3 (18.8)** | **0.462 (0.101-2.100)** | **0.3173** |  |  |
| **BCLC stage** |  |  |  |  |  |
| **B** | **4 (40.0)** | **Referent** |  |  |  |
| **C** | **7 (21.9)** | **0.420 (0.092-1.916)** | **0.2626** |  |  |
| **Beyond up-to-11 criteria** |  |  |  |  |  |
| **No** | **4 (50.0)** | **Referent** |  |  |  |
| **Yes** | **7 (20.6)** | **0.259 (0.052-1.305)** | **0.1016** |  |  |
| **VP stage 4** |  |  |  |  |  |
| **No** | **4 (19.1)** | **Referent** |  |  |  |
| **Yes** | **7 (33.3)** | **2.125 (0.515-8.770)** | **0.2973** |  |  |
| **Occupied ≥ 50% liver volume** |  |  |  |  |  |
| **No** | **5 (55.6)** | **Referent** |  | **Referent** |  |
| **Yes** | **6 (18.2)** | **0.178 (0.036-0.867)** | **0.0327** | **0.173 (0.030-0.994)** | **0.0492** |
| **Both VP4 and ≥ 50%** |  |  |  |  |  |
| **No** | **9 (30.0)** | **Referent** |  |  |  |
| **Yes** | **2 (16.7)** | **0.467 (0.085-2.573)** | **0.3816** |  |  |
| **Extra-hepatic metastasis** |  |  |  |  |  |
| **No** | **10 (35.7)** | **Referent** |  |  |  |
| **Yes** | **1 (7.1)** | **0.138 (0.016-1.220)** | **0.0749** |  |  |
| **AFP (ng/ml)** |  |  |  |  |  |
| **<400** | **6 (28.6)** | **Referent** |  |  |  |
| **≥400** | **5 (23.8)** | **0.781 (0.197-3.106)** | **0.7259** |  |  |
| **Prior locoregional therapy** |  |  |  |  |  |
| **No** | **7 (26.9)** | **Referent** |  |  |  |
| **Yes** | **4 (25.0)** | **0.905 (0.218-3.763)** | **0.8905** |  |  |
| **Firstline systemic treatment** |  |  |  |  |  |
| **No** | **2 (15.4)** | **Referent** |  |  |  |
| **Yes** | **9 (31.0)** | **2.475 (0.452-13.543)** | **0.2960** |  |  |
| **With radiotherapy** |  |  |  |  |  |
| **No** | **4 (14.3)** | **Referent** |  | **Referent** |  |
| **Yes** | **7 (50.0)** | **6.000 (1.353-26.603)** | **0.0184** | **6.138 (1.236-30.494)** | **0.0265** |

Abbreviations: atezo-bev, atezolizumab plus bevacizumab; RT, external beam radiation therapy; ECOG, Eastern Cooperative Oncology Group; ALBI, albumin-bilirubin index; BCLC, Barcelona Clinic Liver Cancer; AFP, alpha-fetoprotein

**Supplementary table 3. Sequential treatments after atezo-bev**

|  | **Group A**  **atezo-bev with RT** | **Group B**  **atezo-bev alone** |
| --- | --- | --- |
| **Any treatment, n (%)†** | **5 (35.7)** | **16 (47.0)** |
| **Systemic treatment** | **4** | **14** |
| **ICI only** |  |  |
| **Pembrolizumab** | **1** |  |
| **Nivolumab plus ipilimumab** |  | **4** |
| **MKI only** |  |  |
| **Sorafenib** | **1** |  |
| **Lenvatinib** | **1** | **2** |
| **Regorafenib** |  | **1** |
| **MKI plus ICI** |  |  |
| **Lenvatinib plus pembrolizumab** | **1** | **2** |
| **Lenvatinib plus nivolumab** |  | **1** |
| **Regorafenib plus nivolumab** |  | **1** |
| **Cabozantinib plus pembrolizumab** |  | **1** |
| **Other** |  |  |
| **Mitoxantrone** |  | **1** |
| **Locoregional therapy** | **3** | **7** |
| **TACE** | **3** | **3** |
| **RT** |  | **4** |
| **HAIC** |  | **1** |

**†****Four patients in Group A and three patients in Group B were still undergoing atezo-bev treatment.**

Abbreviations: ICI, immune check point inhibitor; MKI, multikinase inhibitor; TACE, transcatheter arterial chemoembolization; RT, external beam radiation therapy; HAIC, hepatic arterial infusion chemotherapy

**Supplementary table 4. Predictors for progression-free survival in the highly advanced HCC patients received atezo-bev treatment**

| **Variables** | **All (N=48)** | **Median PFS (95% CI)** | **Crude HR (95% CI)** | **p value** | **Adjust HR (95% CI)** | **p value** |
| --- | --- | --- | --- | --- | --- | --- |
| **Age (years)** |  |  |  |  |  |  |
| **<60** | **19 (39.6)** | **3.3333** | **Referent** |  |  |  |
| **≥60** | **29 (60.4)** | **3.7333** | **1.003 (0.508-1.981)** | **0.9929** |  |  |
| **Gender** |  |  |  |  |  |  |
| **Female** | **13 (27.1)** | **2.7333** | **Referent** |  |  |  |
| **Male** | **35 (72.9)** | **3.7333** | **1.011 (0.471-2.171)** | **0.9780** |  |  |
| **Etiology** |  |  |  |  |  |  |
| **Virus** | **41 (85.4)** | **1.6000** | **Referent** |  |  |  |
| **Non-Virus** | **7 (14.6)** | **3.9667** | **1.397 (0.538-3.628)** | **0.4923** |  |  |
| **ECOG** |  |  |  |  |  |  |
| **0** | **29 (60.4)** | **5.3000** | **Referent** |  |  |  |
| **1 or 2** | **19 (39.6)** | **2.8667** | **1.446 (0.712-2.937)** | **0.3071** |  |  |
| **Modified ALBI grade†** |  |  |  |  |  |  |
| **I or IIa** | **28 (60.9)** | **5.7333** | **Referent** |  |  |  |
| **IIb or III** | **18 (39.1)** | **2.6333** | **1.601 (0.804-3.189)** | **0.1807** |  |  |
| **BCLC stage** |  |  |  |  |  |  |
| **B** | **10 (20.8)** | **19.1667** | **Referent** |  | **Referent** |  |
| **C** | **38 (79.2)** | **2.8667** | **3.297 (1.160-9.377)** | **0.0252** | **2.137 (0.696-6.558)** | **0.1843** |
| **Beyond up-to-11 criteria** |  |  |  |  |  |  |
| **No** | **9 (18.8)** | **3.7333** | **Referent** |  |  |  |
| **Yes** | **39 (81.3)** | **3.3333** | **0.998 (0.452-2.202)** | **0.9954** |  |  |
| **Both VP4 and TO ≥50** |  |  |  |  |  |  |
| **No** | **34 (70.8)** | **5.3000** | **Referent** |  | **Referent** |  |
| **Yes** | **14 (29.2)** | **2.3333** | **2.585 (1.217-5.492)** | **0.0135** | **1.352 (0.576-3.174)** | **0.4887** |
| **Extrahepatic metastasis** |  |  |  |  |  |  |
| **No** | **32 (66.7)** | **5.7333** | **Referent** |  | **Referent** |  |
| **Yes** | **16 (33.3)** | **2.8000** | **2.101 (1.045-4.224)** | **0.0371** | **1.423 (0.681-2.974)** | **0.3484** |
| **AFP (ng/ml)** |  |  |  |  |  |  |
| **<400** | **22 (45.8)** | **7.9333** | **Referent** |  | **Referent** |  |
| **≥400** | **26 (54.2)** | **2.8667** | **2.264 (1.086-4.721)** | **0.0292** | **1.735 (0.815-3.691)** | **0.1529** |
| **Prior locoregional therapy** |  |  |  |  |  |  |
| **No** | **29 (60.4)** | **2.8667** | **Referent** |  | **Referent** |  |
| **Yes** | **19 (39.6)** | **6.0000** | **0.436 (0.209-0.913)** | **0.0276** | **0.617 (0.273-1.396)** | **0.2461** |
| **Firstline systemic treatment** |  |  |  |  |  |  |
| **No** | **14 (29.2)** | **3.4883** | **Referent** |  |  |  |
| **Yes** | **34 (70.8)** | **3.7333** | **1.443 (0.673-3.090)** | **0.3458** |  |  |
| **With radiotherapy** |  |  |  |  |  |  |
| **No** | **34 (70.8)** | **2.8667** | **Referent** |  |  |  |
| **Yes** | **14 (29.2)** | **5.2000** | **0.809 (0.394-1.661)** | **0.5643** |  |  |

**†ALBI missing for 1 patient in atezo-bev alone and 1 patient in atezo-bev with RT**

Abbreviations: HCC, hepatocellular carcinoma; atezo-bev, atezolizumab plus bevacizumab; RT, external beam radiation therapy; ECOG, Eastern Cooperative Oncology Group; ALBI, albumin-bilirubin index; BCLC, Barcelona Clinic Liver Cancer; AFP, alpha-fetoprotein
